# Essential Roles and Regulation of the *Legionella pneumophila* Collagen-Like Adhesin during Biofilm Formation

**DOI:** 10.1371/journal.pone.0046462

**Published:** 2012-09-28

**Authors:** Julia Mallegol, Carla Duncan, Akriti Prashar, Jannice So, Donald E. Low, Mauricio Terebeznik, Cyril Guyard

**Affiliations:** 1 Ontario Agency for Health Protection and Promotion (OAHPP), Toronto, Ontario, Canada; 2 Department of Laboratory Medicine and Pathobiology, University of Toronto, Toronto, Ontario, Canada; 3 Mount Sinai Hospital, Toronto, Ontario, Canada; 4 Cells and System Biology and Department of Biological Sciences, University of Toronto at Scarborough, Toronto, Ontario, Canada; University of Hyderabad, India

## Abstract

Legionellosis is mostly caused by *Legionella pneumophila* (*Lp*) and is defined by a severe respiratory illness with a case fatality rate ranging from 5 to 80%. In a previous study, we showed that a glycosaminoglycan (GAG)-binding adhesin of *Lp*, named Lcl, is produced during legionellosis and is unique to the *L. pneumophila* species. Importantly, a mutant depleted in Lcl (*Δlpg2644*) is impaired in adhesion to GAGs and epithelial cells and in biofilm formation. Here, we examine the molecular function(s) of Lcl and the transcriptional regulation of its encoding gene during different stages of the biofilm development. We show that the collagen repeats and the *C*-terminal domains of Lcl are crucial for the production of biofilm. We present evidence that Lcl is involved in the early step of surface attachment but also in intercellular interactions. Furthermore, we address the relationship between Lcl gene regulation during biofilm formation and quorum sensing (QS). In a static biofilm assay, we show that Lcl is differentially regulated during growth phases and biofilm formation. Moreover, we show that the transcriptional regulation of *lpg2644,* mediated by a prototype of QS signaling homoserine lactone (3OC12-HSL), may play a role during the biofilm development. Thus, transcriptional down-regulation of *lpg2644* may facilitate the dispersion of Lp to reinitiate biofilm colonization on a distal surface.

## Introduction

Legionellosis is a major public health concern in industrialized countries. Manifestations of this disease range from a mild respiratory illness to a severe and rapidly fatal pneumonia [1]. The case fatality rate of legionellosis ranges between 40 to 80% in untreated immuno-suppressed patients but can be reduced from 5 to 30% with appropriate case management [2], [3]. Legionellosis is acquired by inhaling airborne water droplets contaminated with Gram-negative bacilli of the *Legionella* genus [4]. *Legionella* bacteria are found worldwide and can be detected in up to 80% of freshwater sites [5]. Among more than 54 species of *Legionella*, *L. pneumophila* is the major cause of outbreaks (91.5%) and serogroup 1 (*Lp1*) is the predominant serotype isolated from patients (84.2%) [6], [7]. Since human-to-human transfer of the bacteria has not been reported [8], to explain the higher incidence of *Lp1* compared to other *Legionella* species, it has been hypothesized that *Lp1* is more virulent and/or more fit for survival in anthropogenic aquatic environments [9].

While it can replicate within environmental protozoa, another efficient way for *Lp1* to persist and disseminate in man made water systems is to form monospecies and multispecies biofilms [10]–[14]. This mode of living acts as a protective mechanism against a variety of environmental assaults, such as antibiotics or biocide treatments [15]. Therefore, when man-made water systems are contaminated with *L. pneumophila*, the development of biofilm renders water disinfection strategies such as chlorination ineffective [16]. Although it remains unexplored for *Legionella*, in other pathogenic bacteria, the production and development of biofilms *in vivo* promote persistence, colonization of the airways and increases resistance to antibiotics [17]–[19].

The formation of biofilm is a developmental process with multiple steps including cell adhesion, proliferation of sessile cells forming micro-colonies which produce an extra-cellular matrix and maturation into a 3-dimensionnal shape [20]. The last stage is the detachment of bacteria from matrices to reinitiate biofilm colonization on a distal surface [20]. Biofilm formation is a highly intra/inter species regulated process that is controlled and coordinated by a wide range of factors including interconnected signalling molecules such as quorum sensing (QS) autoinducers and 3,5-cyclic diguanylic acid [21] http://www.plospathogens.org/article/info:doi/10.1371/journal.ppat.1000483 - aff1. QS is a density-dependent form of bacterial cell-cell communication that operates via secreted small molecules named autoinducers. Upon reaching a threshold concentration, autoinducers trigger induction or repression of genes involved in biofilm maturation and maintenance to functionally coordinate the behaviour of a bacterial population including the expression of virulence factors [22], [23]. A well characterized QS system is *cqsAS* present in *Vibrio cholerae* and other marine *Vibrio* spp. [24], [25]. The CqsAS is involved in cell density-dependent regulation of virulence and biofilm formation through the synthesis of an α-hydroxyketone autoinducer [26]. Recently, a homologous system, named *lqs* was described in *L. pneumophila* including the *lqsR, lqsA* and *lqsS* gene [27], [28]. *Legionella lqsA* can functionally complement a *V. cholerae cqsA* mutant [28]. Interestingly, the *Pseudomonas aeroginosa* homoserine lactone autoinducer (3OC12-HSL) was recently shown to exert a bacteriostatic activity, and decrease biofilm formation of *Lp1*. These effects are correlated to the transcriptional down-regulation of *lqsR* gene in *Lp1* [29]. This finding suggests a connection between 3OC12-HSL and the uniquely identified QS system in *Lp1*.

Among the matrix associated proteins of biofilms, specific adhesins mediate cell to cell interactions and/or initiate surface attachment. In few cases, the same adhesins also play a role in pathogenesis by allowing the binding of planktonic bacteria to host cells or epithelium [30], [31]. In a previous study, we identified a GAG binding adhesin of *Lp1* designated *Legionella* collagen-like protein (Lcl) [32], [33]. Lcl is produced during legionellosis and is absent from *Legionella* species that are rarely reported in patients and poor biofilm producers. Importantly, a mutant depleted in Lcl is dramatically impaired in biofilm formation. Here, to get insight into the molecular mechanisms used by *Lp1* to produce biofilm, we investigated the molecular functions of Lcl during different stages of the biofilm development. Using recombinant variants, we show that the collagen repeats and the *C*-terminal domains of Lcl are crucial for the production of biofilm. We present evidence that Lcl is involved in the early step of surface attachment but also in intercellular interactions. Furthermore, we address the relationship between the regulation of Lcl gene (*lpg2644*) during biofilm formation and the QS system. We show that Lcl is differentially regulated during growth phases and biofilm formation. Moreover, data suggesting that the regulation of *lpg2644* is QS dependent and plays a role during the late stage of biofilm development are presented. Thus, we propose that the regulation of *lpg2644* gene may participate in the dispersion of bacteria to reinitiate biofilm colonization on a distal surface.

## Materials and Methods

### Chemical, Bacterial Strain and Cultivation

All chemicals were purchased from Sigma-Aldrich (Oakville, ON) unless otherwise noted. Restriction and cloning enzymes were purchased from New England Biolabs (Pickering, ON) and used according to manufacturer recommendations. *Legionella* isolates and plasmid vectors used in this study are listed in Table 1. Lp02 is a streptomycin-resistant thymidine auxotroph derived from Lp01. All mutant strains were made in the Lp02 background. Unless specified, *Legionella* isolates were grown on buffered charcoal yeast extract (BCYE) agar at 37°C+5% CO_2_ incubation for 3–4 days and *Legionella* pre-cultures were grown at 37°C and 100 rpm for 24 h in buffered yeast extract (BYE) broth with 100µg/ml thymidine when necessary. *Escherichia coli* strains and plasmids are listed in Table 1. All *E. coli* strains were cultured in Luria-Bertani medium, antibiotic was added to the media at concentrations of 50 µg/ml kanamycin or 100 µg/ml carbenicillin.

**Table 1:** *Legionella* species, *E. coli* strains and plasmids used in this study.

MR Code	Species	Sg	Designation/Plasmid	Source/Ref
23	*L. pneumophila*	1	Lp02	[65]
36	*L. pneumophila*	1	Lp02/pBH6119-*IcmR*p	[32]
52	*L. pneumophila*	1	Lp02 *Δlpg2644*	[32]
69	*L. pneumophila*	1	Lp02 *Δlpg2644/*pBH6119	[32]
70	*L. pneumophila*	1	Lp02 *Δlpg2644*/pBH6119-*IcmR*p	[32]
73	*L. pneumophila*	1	Lp02/pBH6119	[32]
76	*L. pneumophila*	1	Lp02/p*lpg2644*	[32]
77	*L. pneumophila*	1	Lp02 *Δlpg2644/*p*lpg2644*	[32]
122	*L. pneumophila*	1	Lp02/p*lpg2644 Δ*repeats	[32]
123	*L. pneumophila*	1	Lp02 *Δlpg2644/*p*lpg2644 Δ*repeats	This study
124	*L. pneumophila*	1	Lp02 *Δlpg2644 clpg2644*	[32]
130	*L. pneumophila*	1	Lp02 *Δlpg2644 clpg264/*pBH6119-*IcmR*p	[32]
137	*L. pneumophila*	1	Lp02/p*lpg2644 ΔC-*term	This study
138	*L. pneumophila*	1	Lp02 *Δlpg2644/*p*lpg2644 ΔC-*term	This study
2	*E. coli* TOP10		none	Invitrogen
115	*E. coli* TOP10		pCR2.1- *lpg2644 Δ*repeats	This study
116	*E. coli* TOP10		pBH6119-*IcmR*p-*lpg2644 Δ*repeats	This study
131	*E. coli* TOP10		pCR2.1- *lpg2644 ΔC*-terminal	This study

Sg: serogroup.

### Lcl Detection by Live-cell Confocal Imaging

To detect Lcl at the surface of *L.pneumophila*, GFP-Lp02 and GFP-Lp02 Δ *lpg2644* cells were harvested from broth cultures (OD_600_ = 3.0–3.5) and washed twice with phosphate-buffered saline (PBS). To reduce bacterial movement during *in vivo* imaging, glass coverslips were coated with poly-L lysine according to the manufacturer’s instructions (Sigma-Aldrich, Oakville, ON, Canada). Cells were centrifuged onto the coated coverslips for 5 min at RT (1000×g). Where indicated, bacteria were either incubated with anti-Lcl antibodies alone or both anti-Lcl (1∶50) and anti-Lp1 (1∶250) antibodies (Millipore) diluted in PBS simultaneously. Following 5 min incubation at RT, bacteria were washed twice and labelled with alexa–fluor conjugated secondary antibodies (Molecular probes) as indicated for 5 min on ice. The cells were then moved to a pre-cooled stage and labelled with the inner membrane impermeable FM 4–64 dye (Molecular probes) where indicated. Images were acquired using Quorum confocal spinning disc microscope with a 63×oil immersion objective (Leica DMI6000B stand with a Hamamatsu ORCA-R2 camera). Image acquisition was performed using Metamorph Wave FX software and deconvolution was performed using Volocity 4.3 software (Improvision).

### General DNA Techniques

Total genomic DNAs were purified using QIAamp DNA Mini Kit (Qiagen, Mississauga, ON) and plasmid DNAs were purified using QIAprep Spin Miniprep Kit (Qiagen). DNA was quantified by UV spectrophotometry, and 5 or 10 ng was used as a template for each PCR reaction. Taq DNA polymerase, deoxynucleoside triphosphates, and buffer were used according to recommendations of the manufacturer (Invitrogen, Burlington, ON, Canada). PCR amplifications for cloning were performed using Platinum® Taq DNA Polymerase High Fidelity (Invitrogen). A list of primers used can be found in Table 2. Sequencing reactions were performed using the BigDye® Terminator v3.1 Cycle Sequencing kit purified using the BigDye® X Terminator™ Purification kit, and run on 3130×l Genetic Analyzer (Applied Biosystems, Streetsville, ON). All clones were verified by sequencing.

**Table 2:** Oligonucleotides used for PCR, qRT-PCR, sequencing and cloning.

Code	Primers/probes	Amplified DNA	Sequence (5′ to 3′)
1	*lpg2644* F	*lpg2644*	AGACACGTGTTGAATCCACT
2	*lpg2644* R		CACCAAAAGCAATCCGGCCTCGCA
3	*lpg2644 Xho*I F		AGCTCGAGCAATCCGGCCTCGCAAGCC
4	*lpg2644 Eco*RI R		CGGAATTCCGGGTTGCGAGAGTTGGCTA
5	*lpg2644 Δ*repeat F		GATGACGGCCAAGGTGTGCC
6	*lpg2644 Δ*repeat R		TTGAGGTCCTTGAGGTCCAG
7	*lpg2644 ΔC*-terminal F		TAAGCATGGCAAAACTTCAATTTTGAT
8	*lpg2644 ΔC*-terminal R		TGCAGGCACACCTTGGCCGTCATC
9	*lpg2644 Xba*I F		GGTCATCTAGAGAAATAAAGAATGATACATCGA
10	*lpg2644 Sph*I R		GTGAGCGCATGCGCAAAGCGAATTTATGAACA
11	*lpg2644* F		CTAGGTACCCATGGGCTTGTG
12	*lpg2644* R		TGCATCCCACCATGTGCTT
13	*lpg2644* probe		6FAM- CAAACTCAGATCAAGTTAAT-MGBNFQ
14	*gyrA* F	*gyrA*	GGCGGGCAAGGTGTTATTT
15	*gyrA* R		GCAAGGAGCGGACCACTTT
16	*gyrA* probe		VIC-CATTTCGTTCAGTAACCTG-MGBNFQ

### SDS-PAGE and Immunoblot Analyses of Full Length and Recombinant Fragments of Lcl

Immunoblot analyses were performed with bacterial lysates and/or supernatants harvested from broth cultures at different growth phases, with or without *N*-(3-oxododecanoyl)-l-homoserine lactone (3 OC12-HSL) and from in 2 days and 6 days old biofilms.To obtain exponential (E, OD_600 nm_ 0.5–2.0), post exponential (PE, OD_600 nm_ 2–3.5), mid stationary (MS, OD_600 nm_ 3.5–5.0) and late stationary (LS, OD_600 nm_ >4.5) overnight pre-cultures of Lp02 were adjusted to an OD_600 nm_ of 0.05 in BYE broth and were incubated at 37°C with constant shaking (100 rpm) respectively for 12 h, 18 h, 24 h and 42 h. Cell lysates were prepared by adjusting *Legionella* cultures to OD_600 nm_ of 4.5 and washing twice with chilled 1X PBS. Equal volumes of Laemmli loading buffer containing 2-mercaptoethanol (final concentration 5%) were added to the cells. Supernatants were precipitated using 2, 2, 2-trichloroacetic acid. Pellets were washed twice with chilled acetone and resuspended in Laemmli loading buffer after normalization to the OD_600 nm_ of their corresponding culture. Lysates and supernatants were boiled at 95°C for 10 min prior to loading onto 4–15% linear gradient Tris-HCl gels (Bio-Rad). Sodium dodecyl sulphate-polyacrylamide gel electrophoresis (SDS-PAGE) was performed according to Laemmli [34]. Immunoblotting was performed as described by Towbin *et al.* [35]. SDS- Page Acrylamide gels were stained using Coomassie (SimplyBlue SafeStain, Invitrogen Rockville, Maryland, USA). Visual inspection of SDS-PAGE profiles with normalized secreted fractions revealed similar patterns. For immunoblot analyses, proteins were transferred onto nitrocellulose membranes (Bio-Rad). Bound Lcl (1∶20,000) and GFP (1∶5,000) (Invitrogen) antibodies were detected with peroxidase-linked anti rabbit IgG (1∶160,000).

### Generation of p*lpg2644 Δ*repeats and p*lpg2644 ΔC*-terminal

To remove the collagen-like tandem repeats from *lpg2644* gene of the pCR2.1-*lpg2644* plasmid, 10 ng of plasmid were reverse PCR amplified using primers 5 and 6 (Table 2). Resulting PCR product was blunt end ligated and transformed into *E. coli* TOP10 (Invitrogen). Transformants with pCR2.1-*lpg2644 Δ*repeats were selected by carbenicillin-kanamycin resistance screening on LB agar. The *lpg2644 Δ*repeats fragment was next excised from the plasmid using restriction enzymes *Xba*I and *Sph*I and following gel-purification was subcloned into the *Legionnella* expression shuttle vector pBH6119*-IcmRp* digested with *Xba*I and *Sph*I to generate the plasmid p*lpg2644 Δ*repeats. *E. coli* TOP10 transformed with p*lpg2644 Δ*repeats were selected by carbenicillin resistance screening on LB agar and grown overnight in LB media with 50 µg/ml carbenicillin for plasmid extraction. p*lpg2644 Δ*repeats plasmid was then transformed into Lp02 and Lp02 *Δlpg2644*.

To remove *ΔC*-terminal domain from *lpg2644* gene of the pCR2.1-*lpg2644* plasmid, the same procedure was used with primers 7 and 8 (table 2).

### Static Biofilm Formation Assay

Biofilm assays using polystyrene 96-well plates assays (Costar polystyrene 3361, Corning Incorporated, Lowell MA) were performed according to Piao et *al.* with few modifications [9]. *Legionella* pre-cultures were grown with or without 10 µM 3OC12-HSL for 30 h in broth, adjusted to OD_600 nm_ of 0.2 and 200 µl was used to seed each well in the 96-well microtitre plates. Following incubation at 37°C+5% CO_2_ for 2 or 6 days, the biomass of biofilm was quantified by crystal violet staining assay. Bacteria were stained with 40 µl 0.25% crystal violet in each well for 15 mn, washed 3 times with 200 µl sterile distilled water and were solubilised in 200 µl 95% ethanol at room temperature for 15 min. Absorbance was read at OD_600 nm_ with a microplate reader. Each experiment was repeated three times with six technical replicates for each condition and control.

This assay was slightly modified in order to measure the effect of 3OC12-HSL (Sigma) on the regulation of *lpg2644* gene on biofilm formation. Adjusted pre-cultures were seeded in 96-well microtitre plates and following a 2 h incubation at 37°C+5% CO_2_, with or without 10 µM 3OC12-HSL, wells were washed to remove planktonic cells. Fresh BYE media with or without 10 µM 3OC12-HSL was then added to the sessile cells for 46 h at 37°C+5% CO_2_ and production of biofilm was measured using the crystal violet staining assay described above.

### Confocal Laser Scanning Microscopy

For confocal laser scanning microscopic examination (CLSM) of biofilms, single or mixed bacterial cultures (300 µl) were prepared in Lab-TekII chamber slides (Labtek II, VWR, Rochester, USA) according to the procedure described above. After 3 days of incubation at 37°C+5% CO_2_, supernatants were removed and bacteria were labelled with nucleic acid stain SYTO 62 (Invitrogen) for 30 min at RT. After two washes with sterile distilled water, 4% PFA was added for 15 min followed by one wash with sterile distilled water. The plastic wells were removed from the slide and fluoromount (DAKO north America INC, Carpinteria, USA) was added before placing a coverslip on the gasket and observed by CLSM using a Nikon Eclipse TE2000EZ inverted microscope, 100 X Plan APO oil immersion DIC N2 objective. Image acquisition and post-acquisition processing were performed using EZ-C1 Software Ver. 3.50 and the NIS-elements BR Software Ver. 3.0 for Nikon C1 Confocal Microscopy.

### Attachment Assay of *L. pneumophila* to 96 Polystyrene Microtitre Plate using Quantitative PCR and Confocal Microscopy (CLSM)

Early stage of biofilm formation was examined by measuring the ability of cells to adhere to 96-well (flat bottom) polystyrene microtitre plate (Costar). Assays for attachment were performed using *L. pneumophila* strains Lp02, Lp02 harbouring an empty plasmid (Lp02 pBH6119), Lp02 deleted in *lpg2644* gene (Lp02 *Δlpg2644*) and complemented mutant (Lp02 *Δlpg2644*p*lpg2644*) (Table 1) grown on BCYE agar with ±100 µg/ml thymidine and incubated for 4 days at 37°C+5% CO_2_. Cell suspension was obtained by collecting colonies with a sterile swab and suspending them in 5 ml of BCYE broth. Cell suspension was adjusted to an OD_600 nm_ of 0.1 in 25 ml of BYE broth and was incubated at 37°C with constant shaking (100 rpm). After 24 h of growth, bacteria were harvested and resuspended in BYE at OD_600 nm_ of 2. Two hundred microliters of *Legionella* suspensions of each strain were aliquoted into 96-well microtitre plate (in triplicate) and then incubated for 1.5 h at 37°C+5% CO_2_. After 3 washes in PBS, DNA from adhering cells was purified directly from each well with a DNeasy 96 Blood and Tissue Kit according to manufacturer instructions (Qiagen). Genome copy numbers present in each well were next quantified by quantitative PCR (qPCR) using oligonucleotides and probe to *gyrA* (Table 2, code 14 to 16). Quantitative PCR was performed with the Universal PCR Master Mix (Applied Biosystems) using 400 nM of each specific primer and 200 nM of probe. Amplification and detection of specific products were performed with the ABI Prism 7900 detection system (Applied Biosystems) with samples first incubated at 50°C for 2 min, then at 95°C for 10 min, followed by 40 cycles at 95°C for 15 s, and 60°C for 1 min. Attachment of stained *L. pneumophila* to glass cover slips was measured using confocal microscopy**.** Assays were performed as described above and *L. pneumophila* strains were labelled with nucleic acid stain SYTO 62 (Invitrogen) for 30 min at RT, washed twice and fixed with PFA (4%) for 15 min. After two washes, cover slips were mounted with DAKO and slides were analyzed by CLSM.

### Flow Chamber Experiments

In order to non invasively monitor in real-time the adherence and biofilm formation of *L. pneumophila* under dynamic flow conditions, a continuous-flow cell system was set up (Stovall, life science, Greensboro, NC27497). Each channel (1 mm D×4 mm W×40 mm L) was equipped with bubble traps and flow breaks downstream of a peristaltic pump (IPC-N-16; Ismatec SA). The system was assembled using autoclaved silicon tubing (1 by 3 mm), sterilized with 0.5% sodium hypochlorite overnight, and rinsed excessively with sterile water. A volume of 400 µl of stationary phase grown *Legionella* cultures (OD_600 nm_, 3.0 to 3.5), adjusted to a final OD at 600 nm of 0.2 in fresh BYE was injected into flow chambers. Following 1.5 h of incubation under static condition at RT to allow attachment of cells to the chamber, cells were submitted to a constant flow of 5.5 µl/min at RT for 6 days. Biofilm structures were visualized at 2, 4, 6, 24 h, 2 and 6 days with a Nikon Eclipse TE2000EZ inverted microscope, 100 X Plan APO oil immersion DIC N2 objective, Spectra Physics 488 nm laser.

### RNA Isolation and Quantitative RT-PCR Analysis

E, PE, MS and LS cultures were obtained as described above. To evaluate the impact of the LasQS 3OC12-HSL (Sigma) from *P. aeruginosa* on *lpg2644* transcription, 10 µM 3OC12-HSL was added to MS Lp02 cell suspensions adjusted to an OD_600_ of 0.1 and bacteria were incubated at 37°C with constant shaking (100 rpm) for 24 hr. To prevent RNA degradation, 1 ml of each bacterial suspension was treated with RNAprotect Bacteria Reagent kit (Qiagen) and immediately stored at −20 °C.

To isolate sessile cells, biofilm assays were performed in polystyrene 6-well plates (6 ml/well) (Costar) as described above. After 2 and 6 days of incubation, culture supernatants containing planktonic cells were gently removed and suspensions cells were recovered by centrifugation (5 min, 3000 g, RT). Adhering sessile cells were next detached from the wells by pipetting using RNase free-water (Ambion Life Technologies Inc., Burlington, ON, Canada) and recovered by centrifugation (2 min, 3000 g, RT). Samples were then treated with the RNAprotect Bacteria Reagent kit (Qiagen) and immediately stored at −20 °C.

Total RNA was purified from with the RNeasy kit (Qiagen) and contaminating DNA was removed using the DNA-free kit (Ambion). Concentrations of total RNA were determined with a ND-1000 spectrophotometer (Nanodrop). Oligonucleotides and probes to *lpg2644* (Table 2 code 11 to 13*)* and *gyrA* (Table 2 code 14 to 16) were designed with Primer Express 3.0 software (Applied Biosystems) and purchased from Applied Biosystems. The probes consisted of an oligonucleotide labelled at the 5' end with 6-carboxyfluorescein (6-FAM) as the reporter and at the 3' end with carboxytetramethylrhodamine (TAMRA) as the quencher. Quantitative RT-PCR analysis (qRT-PCR) was performed with the QuantiTect Multiplex RT-PCR Kit according to the manufacturer's instructions (Qiagen). The qRT-PCR mixture contained 400 nM of each gene-specific primer, 200 nM of each probe and 5 ng of RNA template. Amplification and detection of specific products were performed with the ABI Prism 7900 detection system (Applied Biosystems) with samples first incubated at 50°C for 20 min, then at 95°C for 15 min, followed by 40 cycles at 94°C for 45 s, and 60°C for 45s. Standard curves were prepared from genomic DNA of *L. pneumophila* Lp02 (1 to 100000 fg) for each gene to correlate the amount of DNA to cycle thresholds. An R^2^ of 0.99 was obtained for all of the primers pairs that were used in this study. The amount of *lpg2644* RNA was normalized to the quantity of *GyrA* RNA present in each preparation. According to the genoscript transcriptomic database, *gyrA* is not significantly regulated during biofilm formation and various growth phases (http://genoscript.pasteur.fr/). Furthermore, in other Gram-negative species, transcriptomic analyses showed that the expression of *gyrA* is not regulated in presence of 3OC12-HSL [36]. Experiments were performed in triplicate with RNA samples isolated from three independent cultures grown at 37°C and repeated 3 times.

## Results

### Lcl is Expressed at the Cell Surface of *L. pneumophila* and Shows a Cluster Distribution

**Figure 1:**
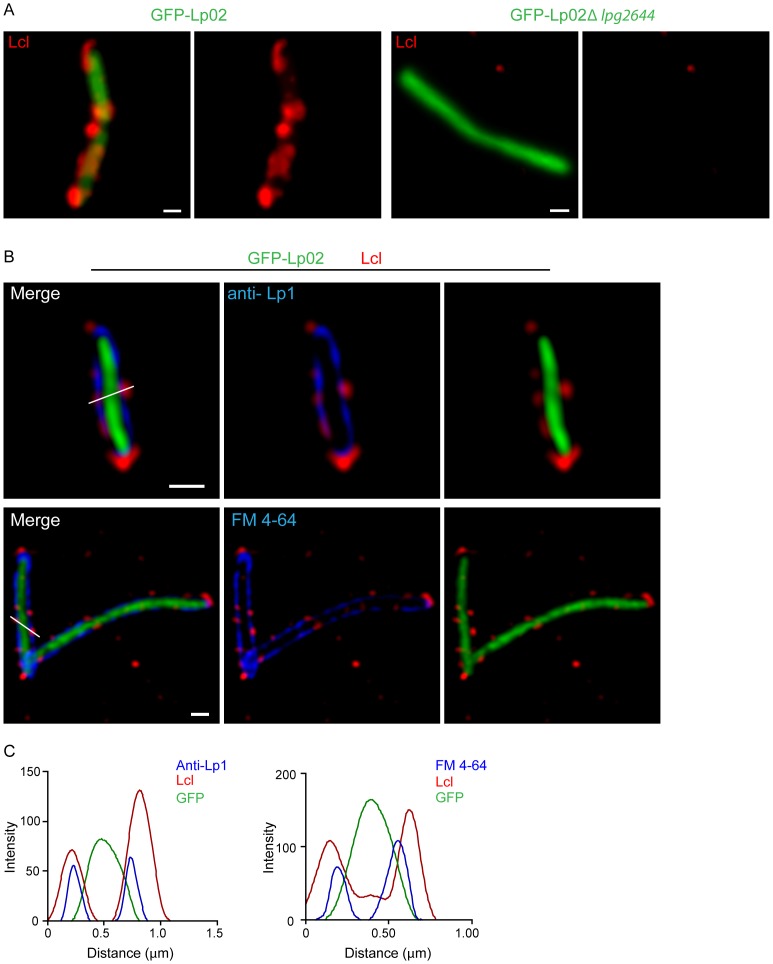
Lcl is expressed at the cell surface of *L. pneumophila* and shows a cluster distribution. Localization of Lcl on the cell surface. (A) GFP-Lp02 (left) and GFP-Lp02 Δ*lpg2644* (right) cells were immuno-labelled using anti- Lcl antibodies followed by alexa 555 (red) conjugated secondary antibodies. Micrographs shown are merged images of 0.2 µm confocal z-stacks. Scale bars, 0.5 µm. (B) Surface localization of Lcl (red) as indicated by its co-localization with membrane labelling anti-Lp1 antibodies (top) and FM 4–64 dye (bottom). Images are confocal xy plane following deconvolution. Scale bars, 0.5 µm. (C) The localization of Lcl (red line) was determined by comparing its fluorescence intensity along the white lines from B, with cytoplasmic GFP (green line) and the outer and inner membrane markers anti-Lp1 (blue line, left) and FM 4–64 (blue line, right), respectively.

In a recent study, we discovered that Lcl promotes the formation of biofilm in *L. pneumophila* [32]. Deletion of Lcl encoding gene (*lpg2644*) dramatically decreased production of *Lp1* biofilm on polystyrene matrix using a static assay. To further characterize the function of Lcl in the production of biofilms in *Lp1*, the cellular localization of Lcl was examined by live imaging using spinning disk confocal microscopy and live cell immuno-fluorescence assays (IFA) of Lp02 GFP expressing cells (see material and methods). Lcl anti-serum reacted with 75% of Lp02 cells. Confocal analysis revealed that Lcl is expressed at the surface of Lp02 cells, albeit not uniformly distributed (Figure 1A). No fluorescence signal was detected with Lp02 *Δlpg2644* and anti-Lcl antiserum (Figure 1A). The cellular distribution of Lcl was further analysed using deconvolved confocal xy planes. To visualize the outer layers of Lp02 by live cell confocal microscopy, inner membranes and outer membrane were labelled with the dye FM4–64 and anti-*Legionella pneumophila* polyclonal antibodies (anti-Lp1), respectively (antibodies are not outer membrane permeable) [37] (Figure 1B). The cellular distribution of Lcl was next analysed by scanning its fluorescence intensity along cross section of the bacteria and compared with the corresponding fluorescence intensities for GFP and either anti-Lp1 or FM 4–64 (Figure 1B and C). This approach demonstrated that Lcl is expressed at the cell surface of *L. pneumophila* and shows a heterogeneous cluster distribution.

### Collagen-like Tandem Repeats and C-terminal of Lcl are Required for Biofilm Formation

**Figure 2:**
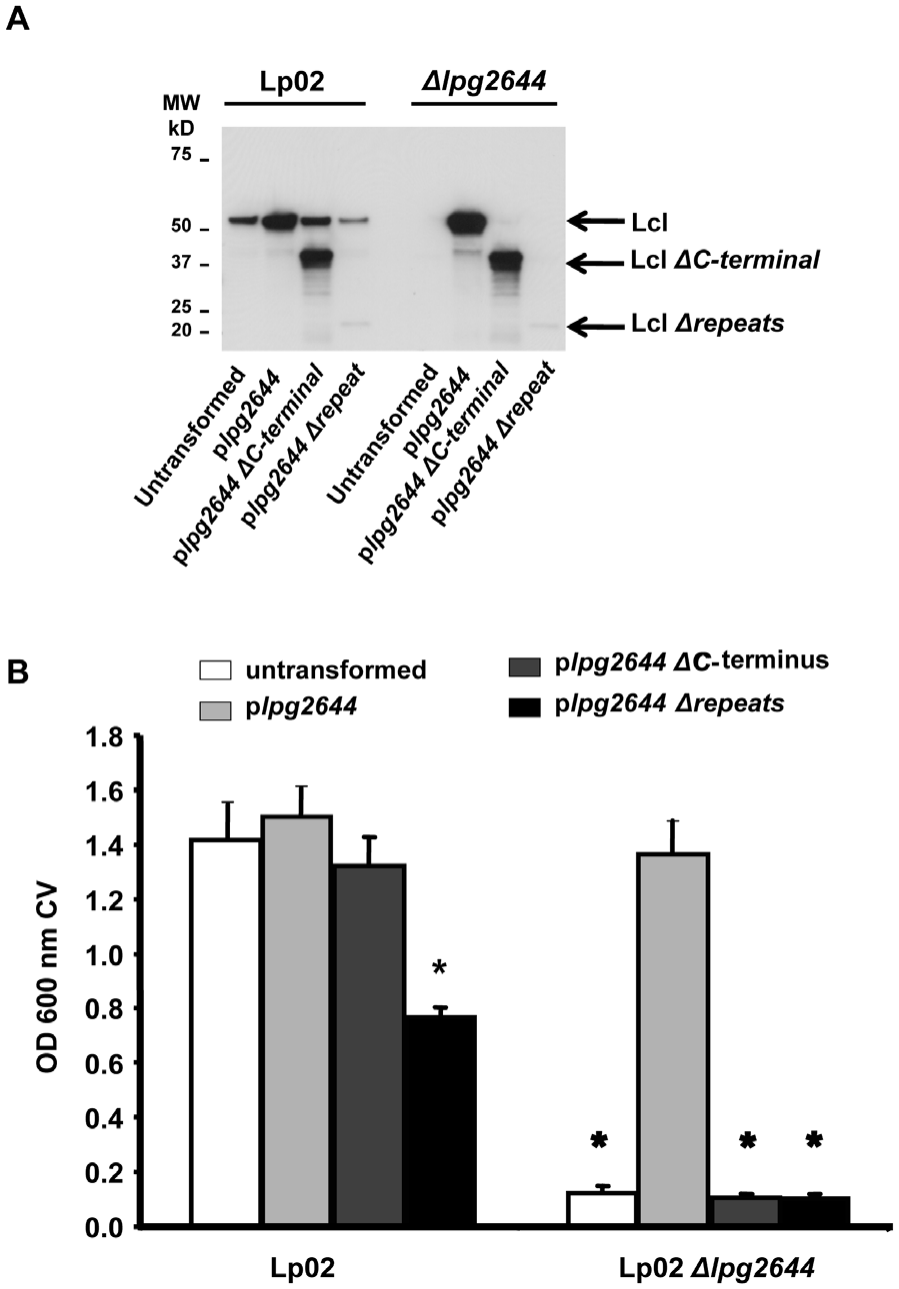
The Collagen-like tandem repeat and the *C*-terminal domains of Lcl are essential for biofilm formation. (A) Anti-Lcl immunoblot analyses against Lp02 and Lp02 *Δlpg2644* lysates untransformed and transformed with p*lpg2644,* p*lpg2644 Δ*repeat and p*lpg2644 ΔC*-terminal. The positions of the molecular size markers are indicated on the left of the blot in kDa. (B) Static biofilm assays with Lp02 and Lp02 *Δlpg2644* producing recombinant variants of Lcl (p*lpg2644 Δrepeat* and p*lpg2644 ΔC*-terminal) and full length recombinant Lcl (p*lpg2644*). * Two-tailed Student’s t-test P-value <0.001 versus Lp02).

The primary sequence of Lcl is divided in 3 structural regions: the *N*-terminal domain including a predicted signal sequence (aa 1–68), a central polymorphic and hydrophilic region comprising collagen-like tandem repeats (aa 69–349) and a *C*-terminal region with no predicted function (aa 350–493). From previous report, it is know that Lcl is an immunogenic protein produced during legionellosis. This immunogenicity is partially driven by Lcl collagen-like tandem repeats [32]. To evaluate the contribution of Lcl collagenous repeats and *C*-terminal region of Lcl in biofilm formation, transcomplementation assays were conducted using replicative expression vectors containing *lpg2644* gene without collagen-like tandem repeats (p*lpg2644 Δ*repeats*)* and *lpg2644* deleted in *C*-terminal region (p*lpg2644 ΔC*-terminal*).* These plasmids were transformed into *L. pneumophila* Lp02 and Lp02 *Δlpg2644* strains (Table 1). Immunoblotting was performed using anti-Lcl antiserum to confirm the synthesis of Lcl *Δ*repeats and Lcl *ΔC*-terminal region in Lp02 and Lp02 *Δlpg2644* transformants (Figure 2A). Anti-Lcl antibodies reacted with a 50-kDa protein in control immunoblot analysis with Lp02 lysates. As expected, no anti-Lcl antibodies reactivity was observed with Lp02 *Δlpg2644*. Immunoblot analysis of all Lp02 transformants showed a dominant band of 50-kDa corresponding to full-length Lcl. A 25 kDa band was weakly recognized by anti-Lcl polyclonal antibodies in Lp02 p*lpg2644 Δ*repeats and Lp02 *Δlpg2644* p*lpg2644 Δ*repeats lysates suggesting that Lcl *Δ*repeats was produced in these strains (Figure 2A). The weak signal observed against Lcl *Δ*repeats is consistent with the high immunogenicity of the tandem repeats and the use of polyclonal antibodies [32]. With Lp02 p*lpg2644 ΔC*-terminal and Lp02 *Δlpg2644* p*lpg2644 ΔC*-terminal lysates, anti-Lcl antibodies reacted with a 32 kDa protein, which corresponds to the predicted molecular mass of Lcl *ΔC*-terminal.

Static biofilm assays were next performed with these strains using the crystal violet method. As expected, after 2 days of incubation at 37°C, Lp02 did form a biofilm while Lp02 *Δlpg2644* did not (Figure 2B). In agreement with previously reported data, the biofilm defect of Lp02 *Δlpg2644* could be restored at the Lp02 level by plasmid containing the full length of Lcl [32]. In contrast, the deficiency could not be rescued by plasmid expressing either a Lcl with a removed repeat region (p*lpg2644 Δ*repeats) or a truncated *C-*terminal (p*lpg2644 ΔC*-terminal). This is suggesting that these 2 domains are essential to the function of Lcl in biofilm formation. Consistent with our previous results, over-expression of Lcl in Lp02 p*lpg2644* did not lead to a significant increase in biofilm formation compared to Lp02 (Figure 2B). Interestingly, while Lp02 p*lpg2644 ΔC-*terminal formed a biofilm similar to Lp02, Lp02 over-expressing Lcl without collagen-like tandem repeat domain induced a highly significant 50% decrease of Lp02 biofilm formation.

### Lcl is Involved in Surface Attachment during Early Step of Biofilm Formation

**Figure 3:**
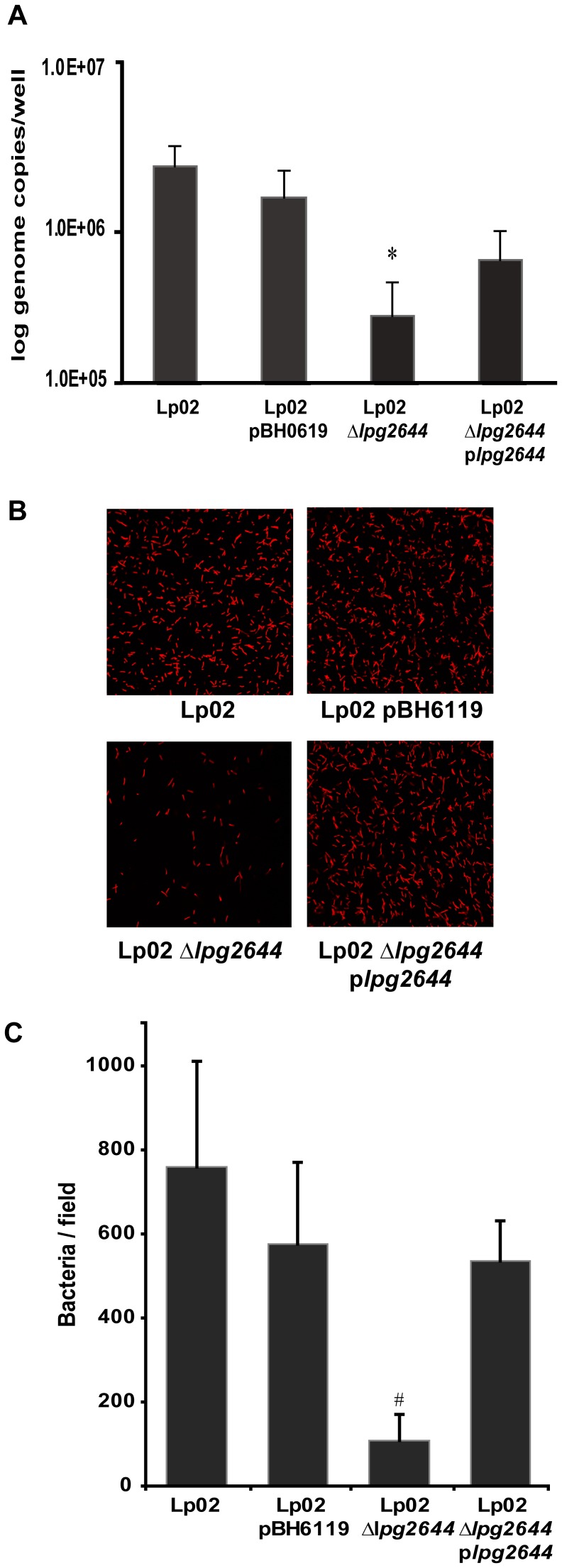
Role of Lcl during the initial attachment step of biofilm formation. The attachment of Lp02, Lp02 pBH6119, Lp02 *Δlpg2644*, and Lp02 *Δlpg2644* p*lpg2644* to the wells of a microtiter plate was measured by (A) qPCR and (B and C) fluorescence microscopy. (A) The binding of Lp02 *Δlpg2644* was significantly reduced compared to the other strains. Values are means+/−s.d. from three individual experiments. * Two-tailed Student’s t-test P-value <0.001 Lp02 *Δlpg2644* vs. all other strains. (B) Comparative analysis of the attachment of SYTO 62 stained *Legionella* strains attached to glass cover slips. (C) Among 10 representative microscopy fields, the average attachment of Lp02, Lp02 pBH6119 and complemented mutant were similar whereas Lp02 *Δlpg2644* showed a marked decrease attachment to surface. * Two-tailed Student’s t-test P-value <0.001.

To determine whether Lcl adhesion is involved in the initial surface attachment of the biofilm formation process, we assessed the adhesion of Lp02, Lp02 pBH6119 (empty replicative plasmid), Lp02 *Δlpg2644* and complemented mutant Lp02 *Δlpg2644* p*lpg2644* to the wells of polystyrene microtitre plates by quantifying attached biomass with qPCR [32]. In control assays, the binding of Lp02 and Lp02 pBH6119 were not significantly different suggesting that the plasmid used in our experiments does not alter the binding of transformed Lp02 (Figure 3A). In contrast, the attachment of isogenic Lp02 *Δlpg2644* to polystyrene was significantly reduced compared to control Lp02 strains (*P≤*0.001, Figure 3A). Although not fully restored, the adhesion of complemented mutant was significantly higher than Lp02 *Δlpg2644* (*P≤*0.05) (Figure 3A). These results were further confirmed by CLSM analysis of SYTO 62 stained *Legionella* strains attached to glass cover slips (Figure 3B). Among 10 representative microscopy fields, the average attachment of Lp02, Lp02 pBH6119 and complemented mutant were similar whereas Lp02 *Δlpg2644* showed a marked decrease attachment to surface (Figure 3C) (P<0.01). These data *s*uggest that Lcl has a relevant role in the initial attachment of *L. pneumophila* to abiotic matrices.

**Figure 4:**
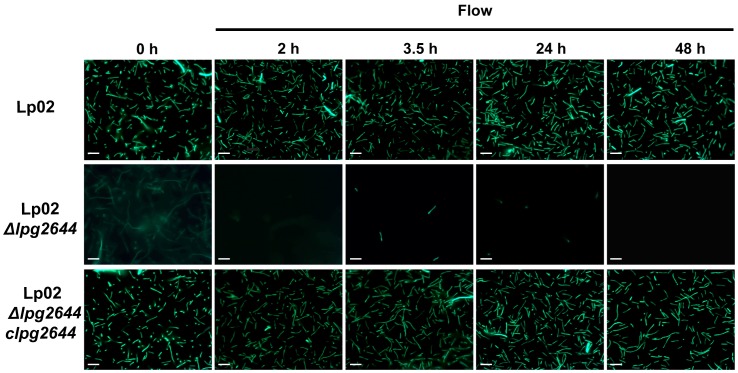
Adherence and persistence of *L. pneumophila* in a continuous-flow chamber system. Fluorescence micrographs of GFP-expressing *L.pneumophila* strains Lp02, Lp02 *Δlpg2644* and chromosomal complemented mutant (Lp02 *Δlpg2644/clpg2644*) in a continuous-flow chamber system operated at 5.5 µl/mn with BYE medium (magnification 100 X). During the initial attachment (0 h), Lp02 and Lp02 *Δlpg2644/clpg2644* strains formed a monolayer on abiotic surface whereas Lp02 *Δlpg2644* was unable to efficiently attach to the flow chambers and detached in a flow dependent manner (2 h to 48 h). Data are representative of three independent replicates. Scale bar, 5 µm.

Bacteria are exposed to shear forces and in response to this stimulus, they tend to attach more firmly to matrices [38], [39]. To determine whether Lcl is likely to be a relevant adhesin in this context, flow cell biofilm assays were conducted. The presence of adhering bacteria was monitored by fluorescence microscopy using bacteria transformed with a plasmid allowing expression of recombinant green fluorescent protein (GFP) (Table 1). In control static biofilm assays, heterologous expression of GFP had no impact on bacterial growth and biofilm formation of Lp02 (data not shown). After inoculation with *Lp1*, flow cell chambers were incubated for 1.5 h (T = 0 h) at RT without flow to allow the cell attachment to the glass substratum. A flow of 5.5 µl/min was next applied for 6 days to allow cell growth and biofilm development. During the initial static incubation, Lp02 and complemented mutant Lp02 *Δlpg2644/clpg2644* strains formed a monolayer of single dispersed cells attached to the surface (Figure 4, T = 0 h). In contrast, although cells in suspension were observed, only few Lcl isogenic mutants seem to attach to the glass surface of the flow chambers (Figure 4, T = 0 h). After the shear force was applied to the chambers, Lp02 and complemented mutant were able to form a monolayer of attached cells (Figure 4, T = 2 to 48 h). This data is consistent with observations of Mampel et *al*. using the same experimental model [40]. In contrast, most Lp02 *Δlpg2644* cells were unable to adhere to the surface and were progressively detached by shear forces (Figure 4). This finding is consistent with our static attachment assays and it suggests that Lcl adhesin is also crucial in the initial attachment of *L. pneumophila* under shear flow during the early step of biofilm formation.

### Role of Lcl in Cell-cell and/or Cell-biofilm Matrix Components during Biofilm Formation

We next hypothesized that in addition to the initial attachment, Lcl could also play a role in cell-cell interactions and/or in the binding of *Legionella* to biofilm matrix components during the late stages of biofilm development. To test this hypothesis, static biofilm assays were conducted with mixed suspensions of Lp02 and Lp02 *Δlpg2644* cells. Co-culture biofilms were formed on chamber slides for 3 days. To differentiate Lp02 from Lp02 *Δlpg2644*, wild type bacteria were transformed with pBH6119-*IcmR*p that allows the expression GFP and Lp02 *Δlpg2644* were transformed with a promoter-less plasmid pBH6119. Plasmids were next swapped to ensure that observed phenotypes were not indirectly linked to the heterologous expression of GFP. To visualise the biofilms, bacteria were labelled with nucleic acid stain SYTO 62 and were analyzed by CLSM.

In control assays, GFP-expressing Lp02 (GFP-Lp02) and Lp02 alone developed similar mature biofilms suggesting that the expression of GFP does not alter the biofilm formation in *Lp1*. In assays with the GFP-expressing mutant (GFP-Lp02 *Δlpg2644*) and the unlabelled mutant, cells were sparsely attached to the surface and did not form micro-colonies (Figure S1).

**Figure 5:**
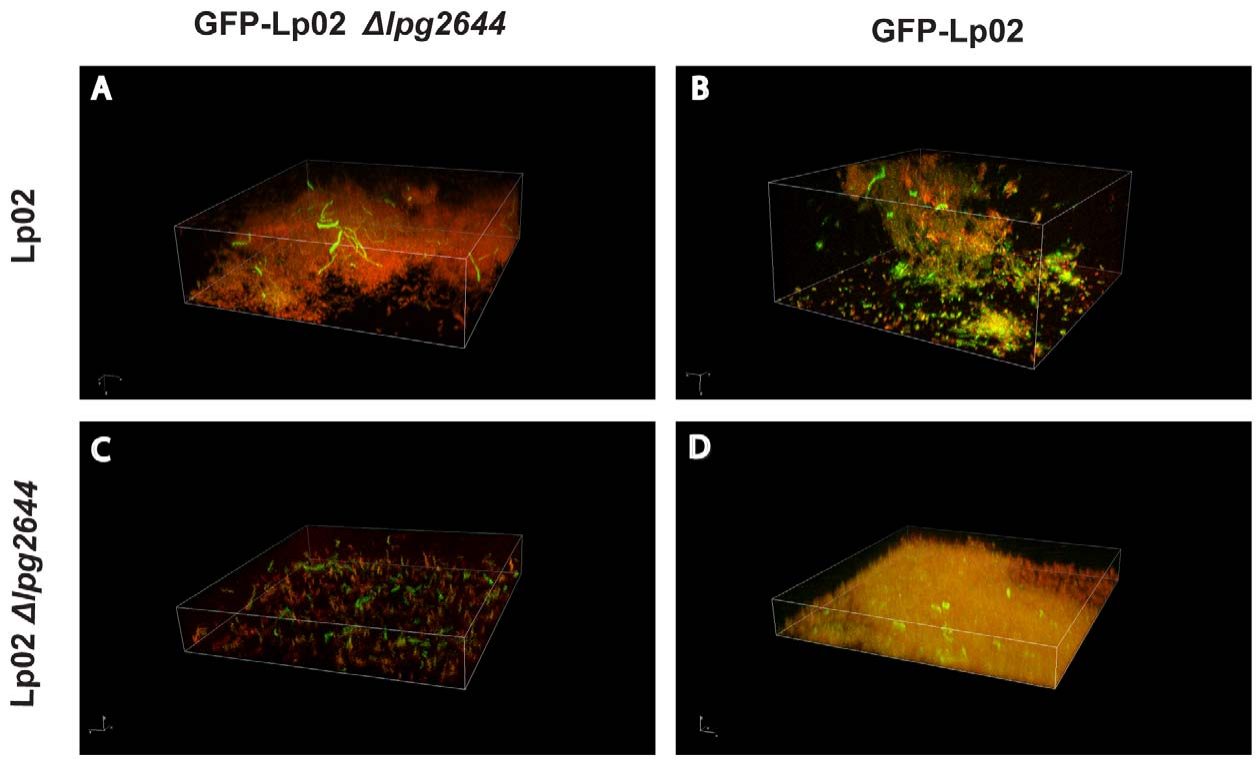
Representative 3D composite images of mixed culture biofilms. Total bacteria were labelled with SYTO62 and one strain of each 1∶1 mixture was labelled with GFP. Confocal scanning laser microscopy (CSLM) micrographs show the distribution of *L. pneumophila* within static biofilms using (A) GFP- Lp02 *Δlpg2644/*Lp02, (B) GFP-Lp02/Lp02, (C) GFP- Lp02 *Δlpg2644/*Lp02 *Δlpg2644*, (D) GFP- Lp02/Lp02 *Δlpg2644.* (Magnification 100 X).

In co-culture assays with Lp02, Lp02 *Δlpg2644* was unable to form micro-colonies and only few individual cells of the mutant were embedded in microcolonies of the wild type strain biofilm (Figure 5A and D). In contrast, in assays conducted with co-cultures of Lp02 and GFP-Lp02, both strains produced a three-dimensional biofilm structure in which individual green bacteria and red bacteria were interspersed homogeneously on the surface and in aggregates (Figure 5B). As expected, when Lp02 *Δlpg2644* and GFP-Lp02 *Δlpg2644* were used, the mutant did not form microcolonies and only sparse cells were observed (Figure 5C). Taken together these results suggest a role of Lcl in cell-cell or cell-matrix component interactions during biofilm development.

### Regulation of Lcl Expression in different Growth Phases and Biofilm Formation

**Figure 6:**
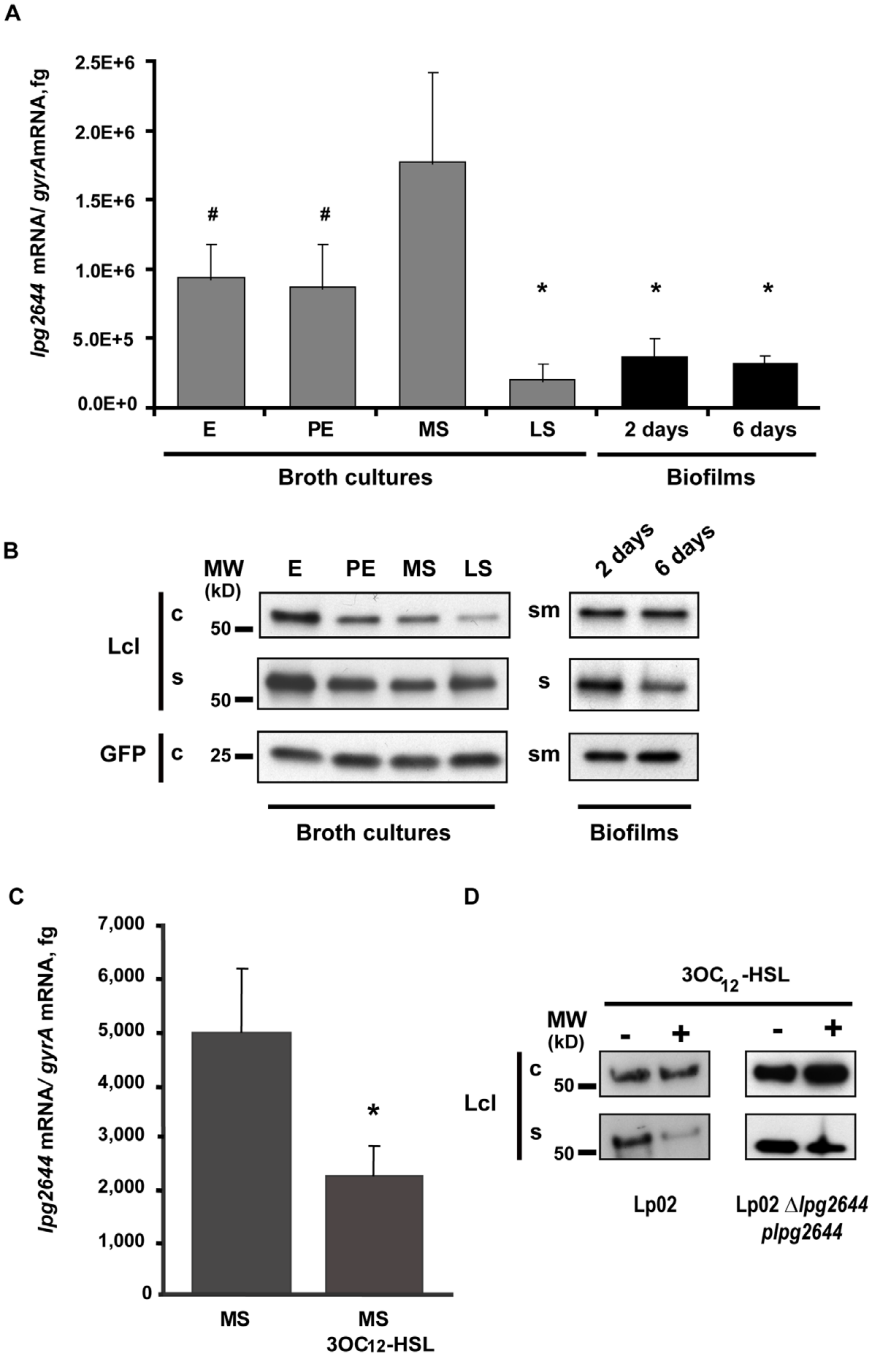
Transcriptional regulation of *lpg2644* and synthesis of Lcl in broth cultures, biofilm and in presence of 3OC_12_-HSL homoserine lactone. Amounts of *lpg2644* transcripts and synthesis of Lcl were respectively measured by (A) qRT-PCR and (B) anti-Lcl immunoblot analysis of cell pellet (c) supernatant (s) and sessile cells/matrix (sm) fractions in Lp02 harvested from exponential (E), post-exponential (PE), mid-stationary (MS), late stationary (LS) broth cultures and biofilms (2 and 6 days old). Quantitative RT-PCR values are means+/−s.d. from three individual experiments. # two-tailed Student’s t-test P-value ≤0.01 versus MS phase, * two-tailed Student’s t-test P-value ≤0.001 versus MS phase. (C) Effect of 3OC12-HSL homoserine lactone on *lpg2644* transcription and (D) Lcl synthesis. Lp02 was grown in absence or in presence of 3OC12-HSL (10 µM) for 24 h. Transcriptional level of *lpg2644* was determined by qRT-PCR analysis. Data are representative of 3 independent experiments.* Student’s t-test P-value ≤0,001 versus untreated Lp02. (D) Synthesis of Lcl was estimated by anti-Lcl immunoblot of cell pellets (c) and supernatant fractions (s) of Lp02 and Lp02 *Δlpg2644* p*lpg2644*. The positions of the molecular size markers are indicated on the left of the blot in kDa.

Coordination of multicellular bacterial behaviors such as biofilm formation frequently involves the transcriptional regulation of virulence and metabolic factors [41]. For *Legionella* bacteria, although few studies have addressed the role of transcriptional regulation and signaling in biofilm formation, there remains limited information available regarding the physiological state and gene expression within sessile cells [42], [43]. Given that Lcl is a major player in biofilm formation, we sought to investigate if Lcl expression is regulated at the transcriptional level. To verify this hypothesis, transcription levels of *lcl* gene were monitored by qRT-PCR using RNA from planktonic cells in broth cultures and from sessiles cells collected from static biofilms (Figure 6A). Planktonic cells were harvested during exponential (E), post exponential (PE), mid stationary (MS), and late stationary (LS) growth phases of broth cultures. RNA was also purified from sessile biofilm cells collected after 2 and 6 days of incubation (Figure 6A). In broth cultures, the relative amount of *lpg2644* transcripts were 2 times lower during the E and PE phase and 8 times lower in LS phase (*P*≤0,001) compared to the MS phase. Interestingly, in 2 or 6 day old-biofilm assays, the amounts of *lpg2644* transcripts were significantly lower than in E, PE and MS phases of the broth cultures (*P≤*0,001) but presented no significant (6 days) or limited differences (2 days, *P*≤0.05) with the LS phase. The largest differences compared to biofilms (2 and 6 days) were observed with MS growth phase bacteria which showed five times more relative amounts of *lpg2644* transcripts (*P*≤0,001) (Figure 6A). This finding was further confirmed with the observation that the amount of *lpg2644* transcripts from planktonic cells recovered from the supernatant of 2 day old biofilms was 1.7 times higher than in sessile cells (*P*≤0,001) (data not shown). These results indicate that the expression of *lpg2644* gene differs significantly during the transition from planktonic cells to sessile cells.

Next, the synthesis of Lcl protein was analysed at different growth phases and in biofilms. For broth cultures, the cells and supernatant were separated by centrifugation, and the presence of Lcl protein in each fraction was assessed by anti-Lcl immunoblot analysis. As shown in Figure 6B and 6D, secreted and cellular Lcl presented the same predicted molecular weight. Approximately 1.4 to 2.9 times less cellular Lcl was produced in LS phase than in E, PE and MS phases, as estimated by densitometry (Figure 6B). The moderate differential transcription observed between E, PE and MS phases by qRT-PCR did not translate into different amount of cellular Lcl by immunoblot analysis. Contrasting with transcriptional analysis data, the amounts of cellular and secreted Lcl in E phase were higher than any of the other growth phases. In the supernatants of PE, MS and LS broth cultures, amounts of extracellular Lcl were similar suggesting that Lcl is stable in the extracellular milieu (Figure 6 B). For biofilms assay analyses, planktonic cells and supernatants were separated from the biofilm biomass (sessile cells and matrix proteins) by collecting non-adhering material from each well. Supernatant fractions (s) were next separated from planktonic cells by centrifugation. The presence of Lcl protein in the sessile cells/matrix (sm) and the supernatant protein fractions was next assessed by anti-Lcl immunoblot analysis (Figure 6B). Although not directly comparable with broth cultures, in 2 or 6 day old-biofilm assays, the amounts of Lcl protein in the sm fractions were similar to the amounts of cellular Lcl in the E, PE and MS phases of the broth cultures (Figure 6B). This result may be explained by encasement of Lcl in the biofilm matrix of static cultures while it is released in the supernatant of broth cultures. Interestingly, the amount of Lcl found in the s fraction was reduced in 6 day old biofilms compared to 2 day old biofilms (Figure 6B). Of note, these fractions contain a mix of secreted proteins from sessile and planktonic cells and can not only be attributed to the biofilms. Taken together, these findings suggest that *lpg2644* gene is moderately up-regulated during the MS phase. Once *Lp1* reaches the LS phase, which is thought to mimic the behavior of bacteria from a mature biofilm, Lcl expression is down-regulated which is in agreement with the decrease transcription of Lcl gene during the late phase of biofilm maturation [44], [45].

### The Transcriptional Regulation of *lpg2644* Gene Plays a Role in Biofilm Development

The QS autoinducer 3OC12-HSL of *P. aeruginosa* which is involved in both intra and inter-species communication has recently been shown to suppress the biofilm production of *Lp1* [29]. Thus, to test whether a QS system is involved in the transcriptional regulation of *lpg2644* during biofilm formation, the Las QS 3OC12-HSL from *P. aeruginosa* was used as a model autoinducer. To rule out a possible growth-suppressive effect of LasQS 3OC12-HSL in our experimental conditions, we first determined the concentration of LasQS 3OC12-HSL that has no effect on *Lp1* growth in broth cultures. A concentration range from 2.5 to 100 µM of 3OC12-HSL was tested and 10 µM was shown to have no bacteriostatic effect on *Lp1* (Figure S2A and B). To evaluate the impact of this autoinducer on *lpg2644* transcription, RNA was extracted from Lp02 broth cultures grown to MS phase in absence and in presence of 10 µM 3OC12-HSL. Transcription levels of *lpg2644* were next determined by qRT-PCR. As shown in Figure 6C, the relative amount of *lpg2644* RNA was 2.2 times lower in presence of 3OC12-HSL compared to untreated cells (P≤0.001). This data suggests that QS autoinducers such as 3OC12-HSL down-regulate the expression of Lcl which is agreement with the down-regulation of *lpg2644* during the late phase of biofilm formation. The synthesis of Lcl were next examined in MS broth cultures treated with 3OC12-HSL (Figure 6D). According to densitometry analyses, the syntheses of Lcl in Lp02 were similar in cellular fractions with 3OC12-HSL and untreated cultures. Interestingly, the main differences between the 2 cultures were observed in the extracellular fractions. When treated with 3OC12-HSL, the amount of extracellular Lcl was reduced by 73% compared to an untreated culture. Of note, this reduction of extracellular Lcl in 3OC12-HSL treated Lp02 was not correlated with the accumulation of cellular proteins. A reduction of protein export by the type II secretion system is known to induce the cellular accumulation of proteins that are normally secreted [46]. As this is not the case here, our data suggest that the synthesis and the secretion of Lcl in Lp02 are both reduced in presence of 3OC12-HSL. In control assays with Lp02 *Δlpg2644* p*lpg2644*, the amount of cellular Lcl was increased in cells treated with 3OC12-HSL. In contrast, the amount of extra-cellular Lcl was decreased in 3OC12-HSL treated cells compared to untreated controls (Figure 6D).

**Figure 7:**
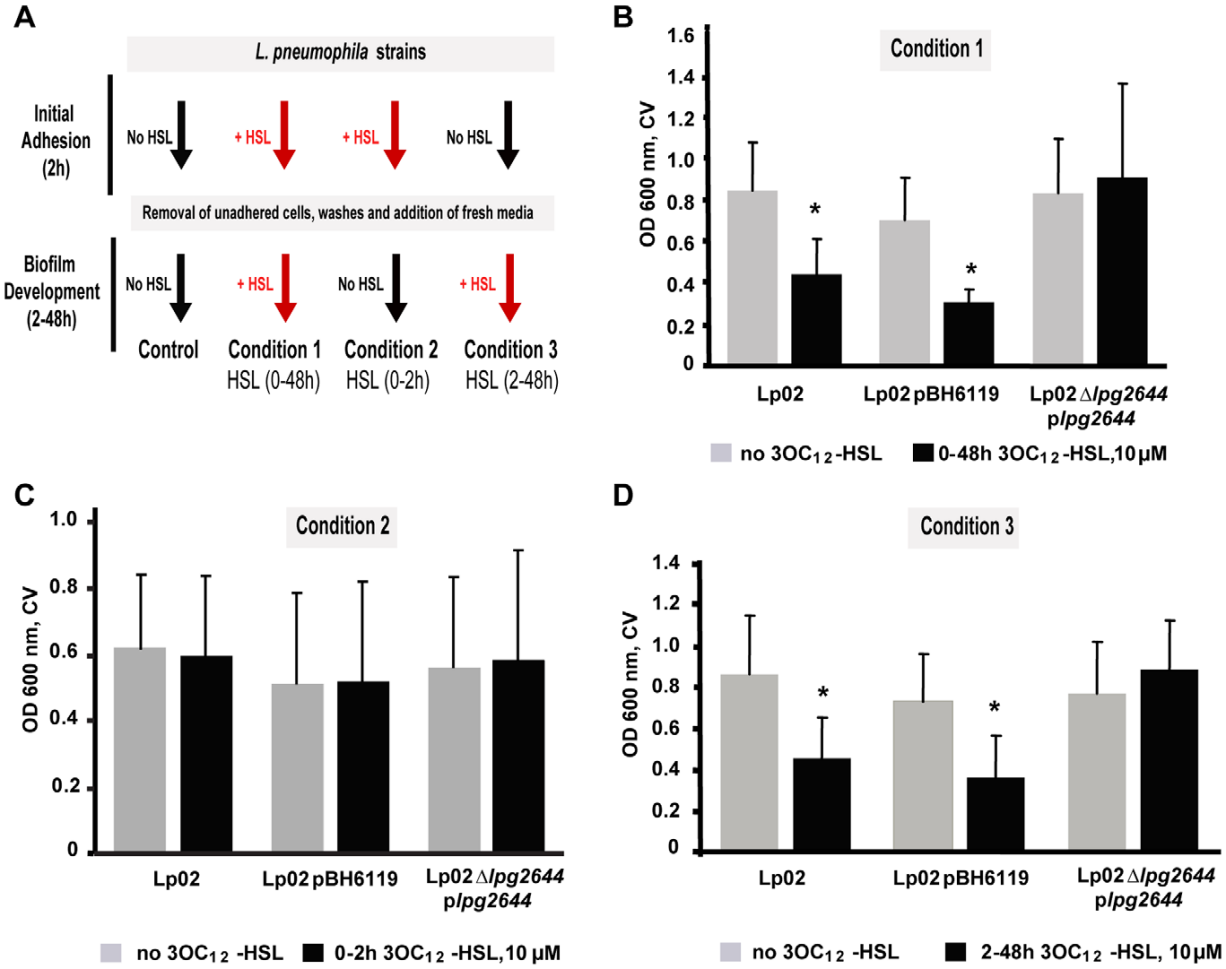
The quorum sensing dependent regulation of Lcl plays a key-role in the control of *L. pneumophila* biofilm development. Flow diagrams indicating the experimental stages when Las QS 3OC12-HSL auto-inducer (HSL) was used and the corresponding static biofilm assays (A). *L. pneumophila* strains Lp02, Lp02 pBH6119 (empty plasmid) and Lp02 *Δlpg2644* p*lpg2644* were exposed to Las QS 3OC12-HSL during 48 h (B), the attachment phase (0–2 h) (C) and biofilm formation (2–48 h) (D). * P value <0.001 no HSL vs+HSL.

This finding raised the possibility that the transcriptional regulation of *lpg2644* may have an impact on *Lp1* biofilm development. Thus, static biofilm assays were conducted in presence or in absence of 3OC12-HSL (Figure 7A). As expected, in presence of 3OC12-HSL, the biofilm formation was significantly impaired in Lp02 (48% inhibition) and Lp02 pBH6119 (42% inhibition) (*P*≤0.001) (Figure 7A and B, Condition 1). In contrast, in trans-complementation assays with a plasmid containing *lpg2644* under the control of the *IcmR* promotor (p*lpg2644*) which is not regulated during biofilm formation (http://genoscript.pasteur.fr/), the biofilm production of Lp02 *Δlpg2644* p*lpg2644* remained unchanged in presence and in absence of 3OC12-HSL (Figure 7A and B, Condition 1). Thus, when Lcl synthesis is under the control of the *IcmR* promoter, the constitutive expression of Lcl is sufficient to maintain biofilm integrity. These results suggest that the transcriptional regulation of Lcl gene through QS signaling plays a key role in *Lp1* biofilm formation.

To further investigate the impact of QS signaling on *lpg2644* transcriptional regulation during the early stage (adhesion) and biofilm development, we used a modified microtitre biofilm assay. Bacterial cells were only exposed to 3OC12-HSL during the initial adhesion phase (Figure 7A, Condition 2) or during the biofilm development phase (Figure 7A, Condition 3). When 3OC12-HSL was only present during the initial adhesion phase (0–2 h), biofilms formed by Lp02, Lp02pBH6119 and Lp02 *Δlpg2644* p*lpg2644* were comparable to controls without 3OC12-HSL (Figure 7C). This result raised the possibility that absence of effect observed on biofilms formation in condition 2 may be due to the short time exposure (2 h) to 3OC12-HSL compared to condition 3 (48 h). To control whether this is the case, similar experiments were performed with bacteria precultured (24 h) with 3OC12-HSL. With this pre-incubation, biofilms formations of Lp02pBH6119 and Lp02 Δ*lpg2644 plpg2644* using condition 2 remained similar (Figure S3). These results suggest that QS regulation of *lpg2644* may only have a minor impact on the initial attachment phase.

In contrast, when bacteria were exclusively exposed to 3OC12-HSL during biofilm development (Figure 7A Condition 3), a marked reduction in biofilm formation was observed with Lp02 (47% inhibition) and Lp02 pBH6119 (50% inhibition) compared to their respective untreated controls (P<0.001) whereas the rescued mutant presented an unaltered biofilm (Figure 7D). These results imply that the deregulation of *lpg2644* gene in the rescued mutant is sufficient to maintain the integrity of biofilms when *Lp1* cells are exposed to 3OC12-HSL. Moreover, these results suggest that the transcriptional regulation of *lpg2644* gene via QS bacterial auto-inducer such as 3OC12-HSL may play a role during biofilm development rather than the adhesion phase of *Lp1*.

## Discussion

The production and colonization of biofilm is considered to play a key role in the environmental dissemination/survival and possibly the pathogenesis of *Legionella pneumophila* [47]. However, little is known about the components and the molecular events that mediate the production of biofilm in these pathogenic environmental bacteria [48]. Only few mediators of *Legionella* biofilm formation have been reported such as FliA [40], a putative twin-arginine protein transport system [49] and the c-di-GMP metabolism [43]. More recently, we reported that Lcl promotes biofilm formation by *Legionella* [32]. In this study, we investigated the immuno-localization, roles and regulation of this surface exposed adhesin during different stages of biofilm formation.

To evaluate the roles of the collagen-like repeats and *C*-terminal domains of Lcl, we transcomplemented a Lcl mutant with variants devoid of these respective domains. None of these variants could rescue the impairment in biofilm formation of the Lcl deletion mutant. Thus, both of these domains contribute to the role of Lcl in biofilm formation. Lcl was found to play a crucial role in the initial attachment of *L. pneumophila* to abiotic matrices during the early step of biofilm development. This was confirmed by our adhesion assays and flow cell experiments showing that attachment of an isogenic mutant Lp02 *Δlpg2644* to polystyrene and glass was significantly reduced compared to Lp02. These findings are correlated with the structural differences observed by CLSM between the biofilms of Lp02 and *Δlpg2644* mutant. They also support the hypothesis that Lcl may participate in cell-cell and/or cell-matrix interactions during biofilm development. In biofilm assays, the few *Δlpg2644* mutant cells bound to the substratum did not produce three-dimensionnal structures in contrast to wild type strain. Moreover, in mixed cultures, the mutant did not form microcolonies and only few Lcl depleted cells were imbedded in Lp02 biofilms. Altogether, these observations suggest a role of Lcl in the anchoring of *L. pneumophila* bacteria to each other or to the biofilm matrix. Given that Lcl is also a secreted protein [50], this finding implies that the secreted protein on its own is not sufficient to rescue the biofilm production impairment of *Δlpg2644* mutant. Thus, Lcl may need to be surface exposed to play a role in biofilm development.

*L. pneumophila* employs a biphasic life cycle to replicate within host cells and to spread to new niches. This process is under the control of a regulatory cascade that responds to a variety of environmental stimuli. Upon entry into stationary growth phase, *L. pneumophila* switches from a replicative (metabolic pathways) to a transmissive (virulent) state, which requires expression of genes involved in virulence, motility, stress resistance and cell division [44], [51]. In this report, we show that the *lpg2644* gene is down regulated in two day old biofilm and during the LS phase compared to the MS growth phase. Taking into consideration that cells grown to the LS phase are known to be metabolically similar to cells of biofilms, this suggests that the pool of newly synthesized Lcl decreases during the late stage of biofilm formation [52]. Therefore, in view of the critical role of Lcl in adhesion and cell-cell or cell-matrix interactions, we propose that the observed transcriptional regulation may participate in the detachment and dispersal of matured *Lp1* biofilm.

In LS phase, the transcriptional down-regulation of *lpg2644* transcription correlated with a reduction of cellular Lcl protein. Interestingly, despite this reduction of cellular Lcl, the amount of extracellular Lcl in the LS phase was similar to other growth phases. This suggests that Lcl may be a relatively stable protein in the extracellular milieu of broth cultures. Moreover, while the transcription of *lpg2644* was found to be down-regulated in biofilms, anti-Lcl immunoblot analyses of biofilm cell fractions revealed higher amount of Lcl compared to cell of LS phase broth cultures. To explain this difference, we are speculating that in biofilms, secreted Lcl is encased in the biofilm matrix and is co-purified with the cellular fraction. This hypothesis is in agreement with the recent discovery of secreted proteins, Bap1 and RbmC, that are selectively retained in the biofilm matrix of *Vibrio cholera* [53].

In Gram-negative bacteria, communication systems (QS sytems) orchestrate important temporal events including synthesis of virulence factors and biofilm formation through the use of autoinducers [54]–[56]. QS bacteria are routinely identified in man-made water systems and it is now recognized that QS systems may play a role in the regulation of environmental biofilm production [57]. In multispecies biofilms, quorum sensing also mediates interactions between bacterial species such as *L. pneumophila* and *P. aeruginosa*. Two dependent QS systems have been described in *P. aeruginosa*: Las and Rhl [58]–[61]. The Rhl-based QS is regulated by *N*-butanoyl- L-homoserine lactone (C_4_-HSL) produced by RhlRI whereas the Las-based QS is regulated by the autoinducer 3OC12-HSL produced by LasRI. *P. aeruginosa* requires the *lasI* gene product (3OC12-HSL) in order to develop a differentiated biofilm and 3OC12-HSL is believed to regulate the complex architecture observed in mature biofilm [55]. Las QS 3OC12-HSL is also involved in inter-species interactions and it has been shown to suppress the growth and the biofilm formation of *L. pneumophila* [29].

Here, we show that a non-growth-suppressive concentration of 3OC12-HSL down-regulates the transcription of *lpg2644* and the secretion of Lcl in MS phase. Interestingly, the biofilm production of a complemented mutant producing Lcl under the control of a constitutive promoter is not impaired in the presence of 3OC12-HSL. This result implies that the constitutive expression of Lcl is sufficient to maintain the biofilm integrity of Lp1 and suggests that the QS regulation of Lcl may play a key role in the course of the biofilm development/maturation of this environmental pathogen. In addition, we uncover that the QS 3OC12-HSL transcriptional regulation of *lpg2644* is preferentially involved after the initial adhesion stage of biofilm development. This result is agreement with data from Davies *and al*. showing that QS 3OC12-HSL participates exclusively in the late phase of biofilm maturation [52], [55].

In *L. pneumophila*, *α*-hydroxyketone (AHK) of the Lqs system is the only QS autoinducer that has been described to date [27], [28]. Host cell interactions are under the control of a complex virulence regulatory network including the *lqs* gene cluster (*lqsA*-*lqsR*-*hdeD*-*lqsS*) [27]. Although it remains unknown if this cluster regulates biofilm production in *Legionella*, it is homologous to the *Vibrio cholerae cqsAS* quorum sensing system 1 which is involved in cell density-dependent regulation of virulence and biofilm formation [24], [25], [62]. *Vibrio* spp. produce three distinct chemical classes of autoinducers: furanosyl borate diester AI-2, N-acyl-L-homoserine lactones (AHLs) and α-hydroxyketones (AHKs). In the Lqs system, the *lqs* gene cluster includes the *lqsR* gene that is flanked by *lqsA* and *lqsS* encoding respectively an autoinducer synthase LqsA and a sensor kinase LqsS. LqsR is a putative response regulator that promotes the induction of transmissive traits including motility and virulence (phagocytosis, intracellular replication and cytotoxicity) and inhibits entry into the replicative growth phase. Globally, this two-component system regulates the expression of a 133 kb genomic island. The auto-inducer synthase LqsA only regulates few genes whereas LqsS regulates the expression of 93 genes suggesting that LqsS responds to additional QS molecules than the α-hydroxyketone of LqsA [27], [28]. In comparative transcriptome analyses, the gene regulation patterns of *ΔlqsS* and *ΔlqsR* mutants showed a strong correlation which suggests that they share a common signal transduction pathway. Among these genes, *lpg2644* was shown to be up-regulated 4.27 times in the absence of Lqs system [63], [64]. Lcl is secreted by the type II secretion system, a secretion pathway that partially relies on the Sec cytoplasmic membrane translocation system [50]. Interestingly *sec* genes were also found to be up-regulated in a *Legionella lqs* quorum sensing mutant [64]. Taking into account that the amount of secreted Lcl was reduced after treatment with HSL and in aging biofilms, these findings suggest that the synthesis and the secretion of Lcl may be co-regulated by the quorum sensing. More work is necessary to evaluate the relative contributions of the regulations of *lpg2644* transcription and Lcl secretion in *Lp1* biofilm formation.

The 3OC12-HSL was recently shown to regulate the transcription of *lqsR* [29]. Although it remains unknown if LqsS responds to homoserine lactones, considering that our study reveals that *lpg2644* is also downregulated by 3OC12-HSL, it is tempting to speculate that *Lp1* responds to QS signaling through at least part of this signal transduction pathway by regulating the expression of Lcl and the production of biofilm. In view of the data presented in this report, it would be interesting to further investigate the role of Lqs system on *lpg2644* transcriptionnal regulation by testing the effect of spent media or purified *Legionella* α-hydroxyketone on Lp02 and by measuring the impact of LqS overexpression on biofilm formation and *lpg2644* transcription.

In summary, we have revealed that Lcl plays a crucial role in biofilm formation during the attachment phase but also in cell-cell interactions phase and/or cell-matrix component interactions where it participates in the three-dimensional structure of the biofilm. Active detachment from the substratum is a physiologically regulated event of biofilm development and our results suggest that homoserine lactone may play a role in detachment during the dispersal phase. This is in agreement with observed down-regulation of Lcl in 2 and 6 day old-biofilms and after incubation of *Lp1* with 3OC12-HSL. Although additional experiment are necessary to confirm this hypothesis, this suggests that homoserine lactones like 3OC12-HSL can stimulate the dispersion of Lp1 biofilm by down-regulating the synthesis of attachment factors such as Lcl. While few studies have addressed the transcriptional regulation/signaling of *Lp1* biofilm, this report is the first to address the role and regulation of a matrix-associated protein of *Lp1* biofilm. In the long term, these findings may lead to the design of alternative disinfection strategies that could more efficiently prevent colonization of man-made water systems by *Legionella*.

## Supporting Information

Figure S1 **Three-dimensional views of biofilm assays visualized by CLSM.** Biofilm assays were performed with (A) GFP-Lp02, (B) GFP- Lp02 *Δlpg2644*, (C) Lp02 stained with Syto 62 and (D) Lp02 *Δlpg2644* stained with SYTO62. All micrographs were taken at 3 days. Single culture of GFP-Lp02 or Lp02 stained with SYTO 62 developed a mature biofilm, whereas assays with GFP- Lp02 *Δlpg2644* or SYTO62 stained Lp02 *Δlpg2644* show isolated cells that did not form micro-colonies.
(TIF)

Figure S2 **A concentration of 10 µM of 3OC_12_-HSL does not affect the growth of** ***L. pneumophila*** **in broth pre-cultures and in biofilm assays.** (A) *L. pneumophila* was grown in BYE medium with (red square) or without (black diamond) 10 µM 3OC_12_-HSL at 37°C with constant shaking. Bacterial growth was determined by measuring the optical density at 600 nm. (B) Growth of *L. pneumophila* during biofilm formation after 48 h of incubation at 37°C with or without 3OC_12_-HSL (10 µM). Bacterial growth was determined by measuring the optical density at 600 nm.
(TIF)

Figure S3 **After a 24** **h pre-incubation with 3OC12-HSL, the quorum sensing dependent regulation of Lcl does not have an impact on the attachment phase (0–2** **h) of biofilm development.** Pre-cultures (24 h) of *L. pneumophila* strains Lp02, Lp02 pBH6119 (empty plasmid) and Lp02 *Δlpg2644* p*lpg2644* with 3OC12-HSL were used in static biofilm assays under the condition 2 described in Figure 7 (attachment phase, 0–2 h).
(TIF)
