# Supplementary figures and images for: Essential Roles and Regulation of the Legionella pneumophila Collagen-Like Adhesin during Biofilm Formation
Source: PLoS One. 2012 Sep 28;7(9):e46462. doi: 10.1371/journal.pone.0046462 (PMC3460888; doi:10.1371/journal.pone.0046462)

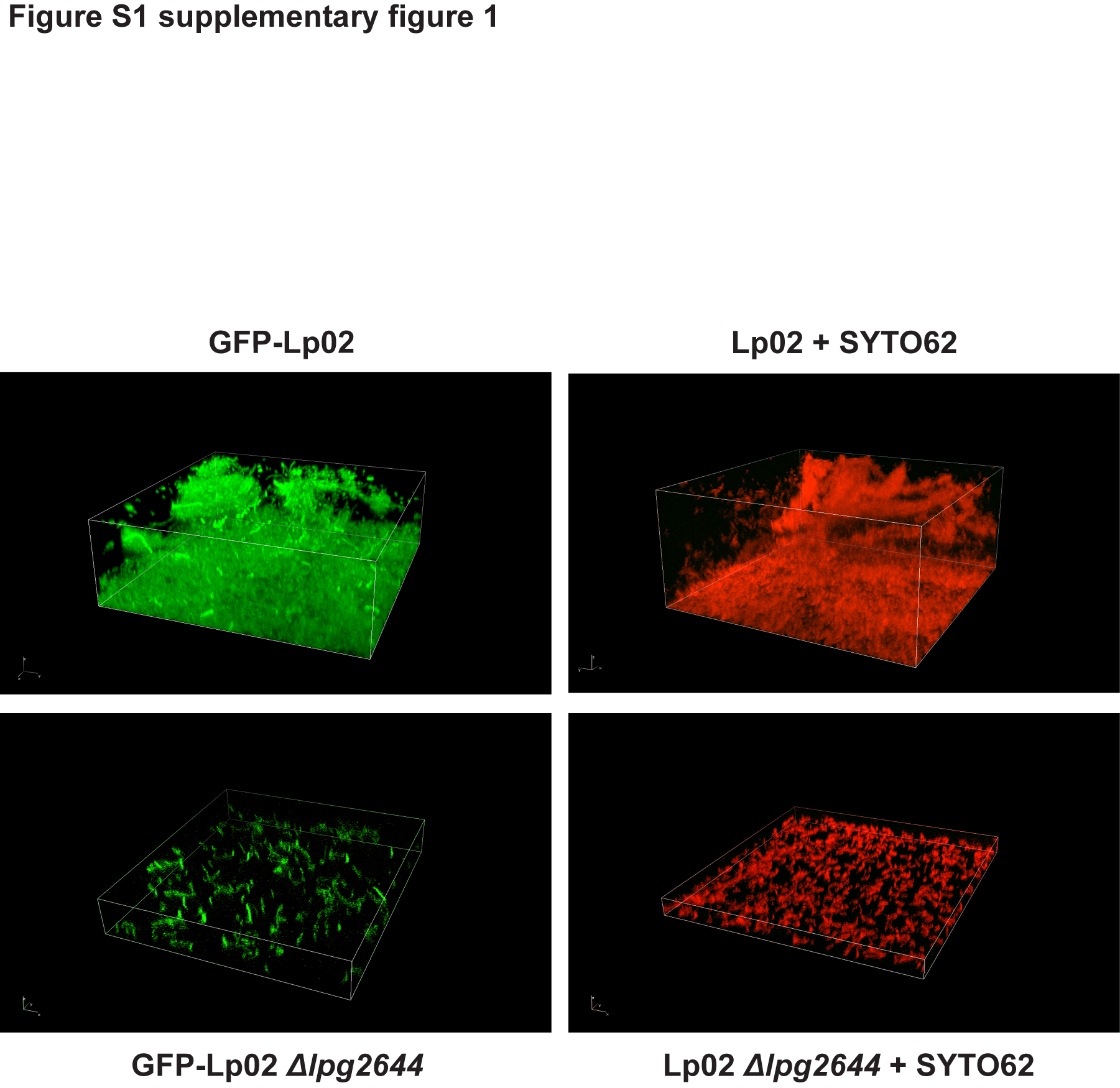

Supplement: Figure S1 — Three-dimensional views of biofilm assays visualized by CLSM. Biofilm assays were performed with (A) GFP-Lp02, (B) GFP- Lp02 Δlpg2644, (C) Lp02 stained with Syto 62 and (D) Lp02 Δlpg2644 stained with SYTO62. All micrographs were taken at 3 days. Single culture of GFP-Lp02 or Lp02 stained with SYTO 62 developed a mature biofilm, whereas assays with GFP- Lp02 Δlpg2644 or SYTO62 stained Lp02 Δlpg2644 show isolated cells that did not form micro-colonies. (TIF) [file pone.0046462.s001.tif]

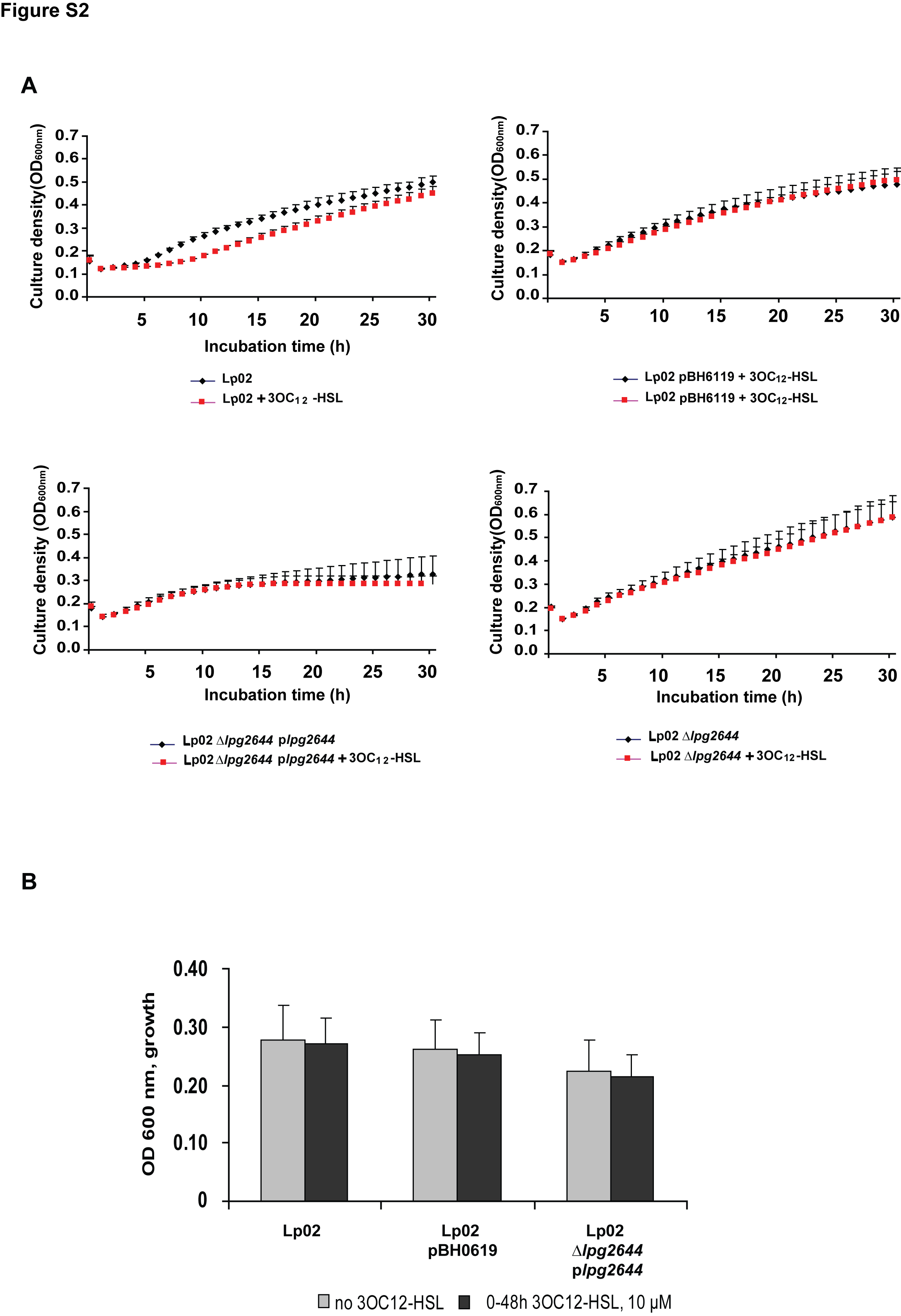

Supplement: Figure S2 — A concentration of 10 µM of 3OC12-HSL does not affect the growth of L. pneumophila in broth pre-cultures and in biofilm assays. (A) L. pneumophila was grown in BYE medium with (red square) or without (black diamond) 10 µM 3OC12-HSL at 37°C with constant shaking. Bacterial growth was determined by measuring the optical density at 600 nm. (B) Growth of L. pneumophila during biofilm formation after 48 h of incubation at 37°C with or without 3OC12-HSL (10 µM). Bacterial growth was determined by measuring the optical density at 600 nm. (TIF) [file pone.0046462.s002.tif]

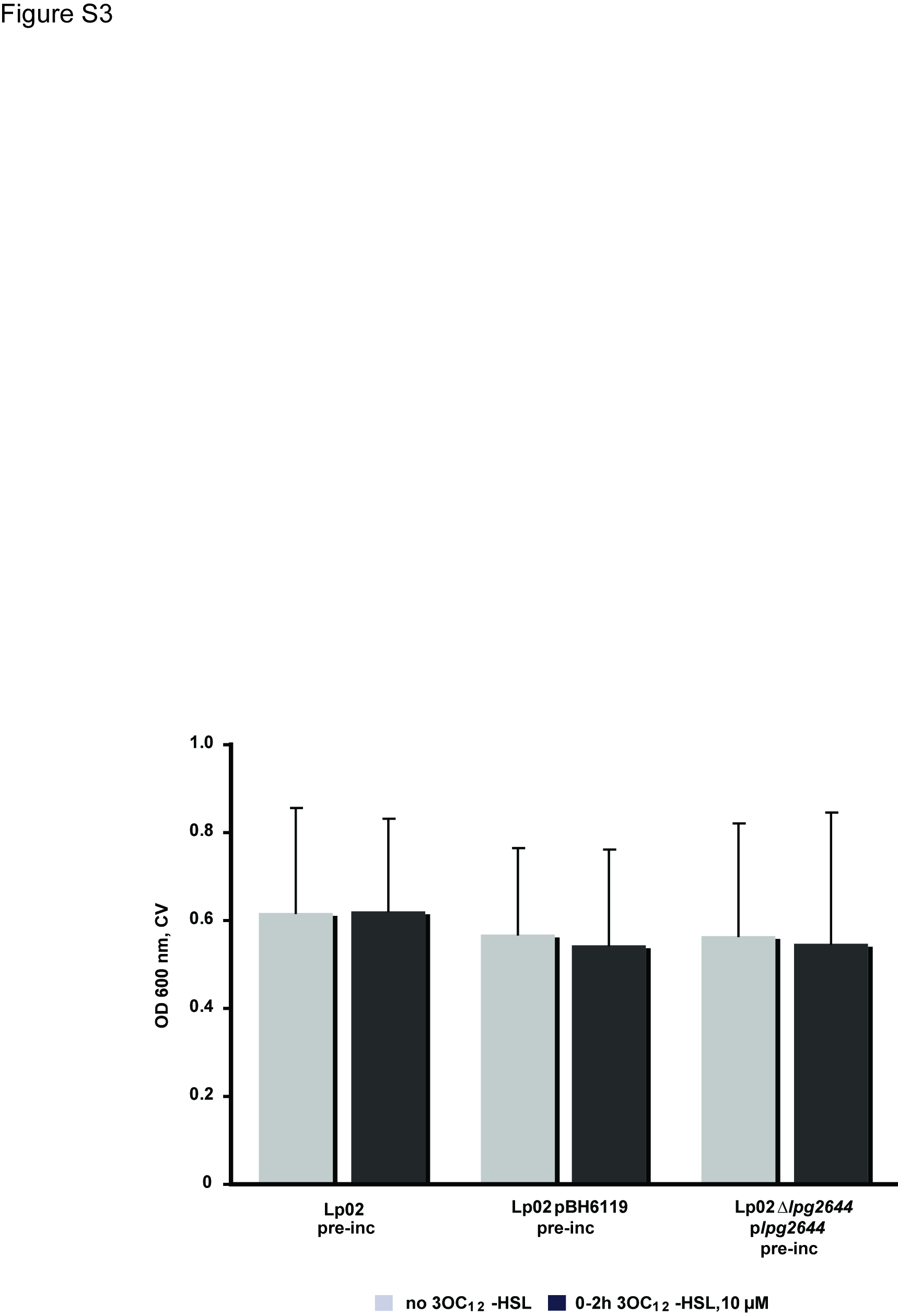

Supplement: Figure S3 — After a 24 h pre-incubation with 3OC12-HSL, the quorum sensing dependent regulation of Lcl does not have an impact on the attachment phase (0–2 h) of biofilm development. Pre-cultures (24 h) of L. pneumophila strains Lp02, Lp02 pBH6119 (empty plasmid) and Lp02 Δlpg2644 plpg2644 with 3OC12-HSL were used in static biofilm assays under the condition 2 described in Figure 7 (attachment phase, 0–2 h). (TIF) [file pone.0046462.s003.tif]
